# Registration Accuracy and Quality of Real-Life Images

**DOI:** 10.1371/journal.pone.0040558

**Published:** 2012-07-19

**Authors:** Wei-Yen Hsu

**Affiliations:** Department of Information Management, National Chung Cheng University, Minhsiung Township, Chiayi County, Taiwan; Institute of Psychology, Chinese Academy of Sciences, China

## Abstract

**Background:**

A common registration problem for the application of consumer device is to align all the acquired image sequences into a complete scene. Image alignment requires a registration algorithm that will compensate as much as possible for geometric variability among images. However, images captured views from a real scene usually produce different distortions. Some are derived from the optic characteristics of image sensors, and others are caused by the specific scenes and objects.

**Methodology/Principal Findings:**

An image registration algorithm considering the perspective projection is proposed for the application of consumer devices in this study. It exploits a multiresolution wavelet-based method to extract significant features. An analytic differential approach is then proposed to achieve fast convergence of point matching. Finally, the registration accuracy is further refined to obtain subpixel precision by a feature-based modified Levenberg-Marquardt method. Due to its feature-based and nonlinear characteristic, it converges considerably faster than most other methods. In addition, vignette compensation and color difference adjustment are also performed to further improve the quality of registration results.

**Conclusions/Significance:**

The performance of the proposed method is evaluated by testing the synthetic and real images acquired by a hand-held digital still camera and in comparison with two registration techniques in terms of the squared sum of intensity differences (SSD) and correlation coefficient (CC). The results indicate that the proposed method is promising in registration accuracy and quality, which are statistically significantly better than other two approaches.

## Introduction

Image registration is a fundamental technology in a variety of fields and has been extensively investigated over the past few decades. It has been applied to many areas, such as medical image analysis, surveillance operations, video representation and retrieval, remote sensing, and consumer device, with different registration techniques and performance requirements [1]–[13]. It is mainly the process of spatially registering acquired images so that corresponding features or pixels on them are consistent in geometry. A common registration problem for the application of consumer device is to align all the acquired image sequences into a complete scene. Image alignment requires a registration algorithm that will compensate as much as possible for geometric variability among images. However, images captured views from a real scene usually produce different distortions. Some are derived from the optic characteristics of image sensors, and others are caused by the specific scenes and objects. In general, we would make some reasonable assumptions to develop a fast algorithm for real time applications in the fields of consumer device. That is, there are no moving objects in the scenes when capturing images, and the images are acquired in short time intervals.

Another important issue for image registration is to determine the transformation model. Depending on the chosen type of spatial transformation, the parameter number of registration model that is required would be decided. The rigid transformation model, which preserves relative distances of points, estimates the translation and rotation, whereas the affine model [14] estimates the rigid transformation parameters and the scale factor. The affine transformation preserves collinearity. That is, parallel lines are transformed into parallel lines, and the ratios of distances are preserved along parallel lines. In addition, a more complex transformation model, perspective projection [15], takes more parameters into account. It considers not only affine transformation but also the transformations of panning and tilting. The transformation model of perspective projection is estimated to apply to the images captured from a consumer device, such as a hand-held digital still camera or a CMOS image sensor.

Moreover, a registration algorithm usually minimizes a cost function that is a combination of an objective function and smoothness constraint [16]–[19]. There are various algorithms that iteratively minimize the surface distance in order to linearly align two regions, such as iterative closest point algorithms.

In this study, we propose an analytic differential approach to achieve fast and robust point matching. A wavelet-based method is used to extract features and discard the noise in multiscale at the same time. It then speedily evaluates the spatial correspondence and geometrical transformation between two point sets with different sizes. It is robust to noise and tolerant to distortion caused by chromatic aberration and geometry discrepancy. Finally, a feature-based modified Levenberg-Marquardt algorithm (FMLM) is used to further refine registration results and speedily obtain subpixel accuracy because of its feature-based and nonlinear characteristic. Furthermore, we also take vignetting artifacts and color/luminance differences into account so as to achieve registration results in high quality.

**Figure 1:**
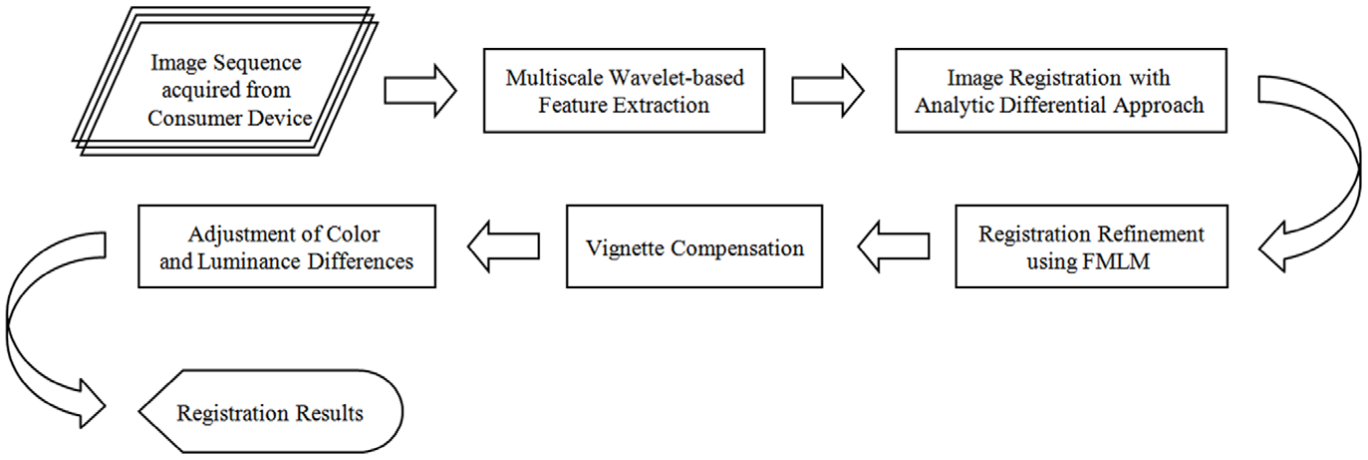
Flowchart of the proposed method. It consists of multiscale wavelet-based feature extraction, image registration with analytic differential approach, registration refinement using FMLM, vignette compensation, and adjustment of color and luminance differences.

The paper is organized as follows. Section 2 describes the proposed method in detail. In Section 3, experimental results and a discussion for some validation examples are presented. Finally, the conclusion and future work is given in Section 4.

**Figure 2:**
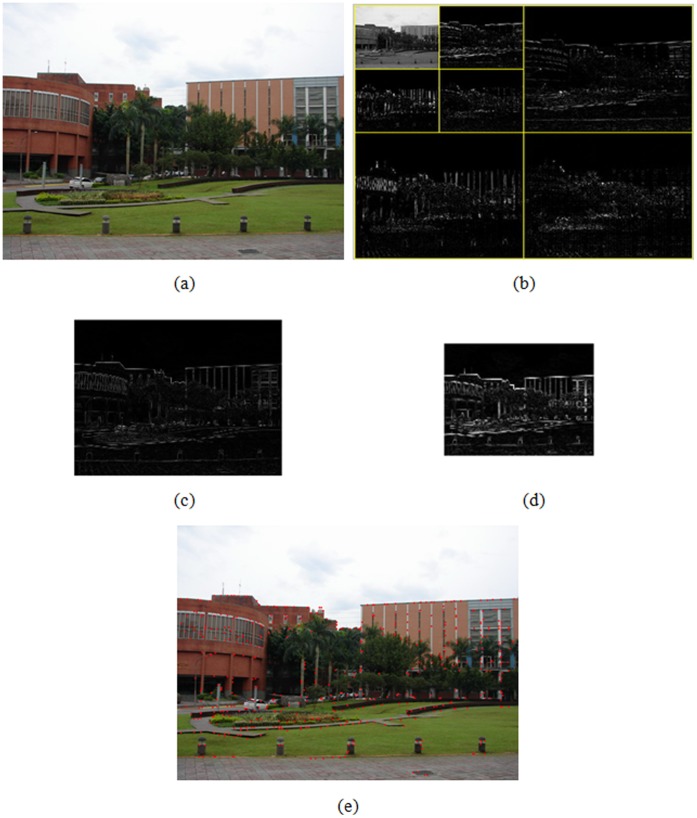
Procedure of feature extraction. (a) A test image, (b) 2D 2-level wavelet decomposition of test image, (c), (d) gradient modului at level 1 and 2, respectively, (e) result of feature point extraction.

## Methods

The proposed method consists of feature extraction, image registration, registration refinement, and vignette compensation and color difference adjustment. First, feature points are extracted by wavelet-based edge correlation with large responses in multiscale. An analytic differential approach for robust point matching (RPM) algorithm is then proposed to achieve fast convergence and complete image registration of perspective projection as well. Next, a FMLM method is proposed to further obtain subpixel accuracy. Finally, vignette compensation as well as the adjustment of color and luminance differences is performed to enhance the quality of registration results. The flowchart of the proposed method is illustrated in Fig. 1.

**Figure 3:**
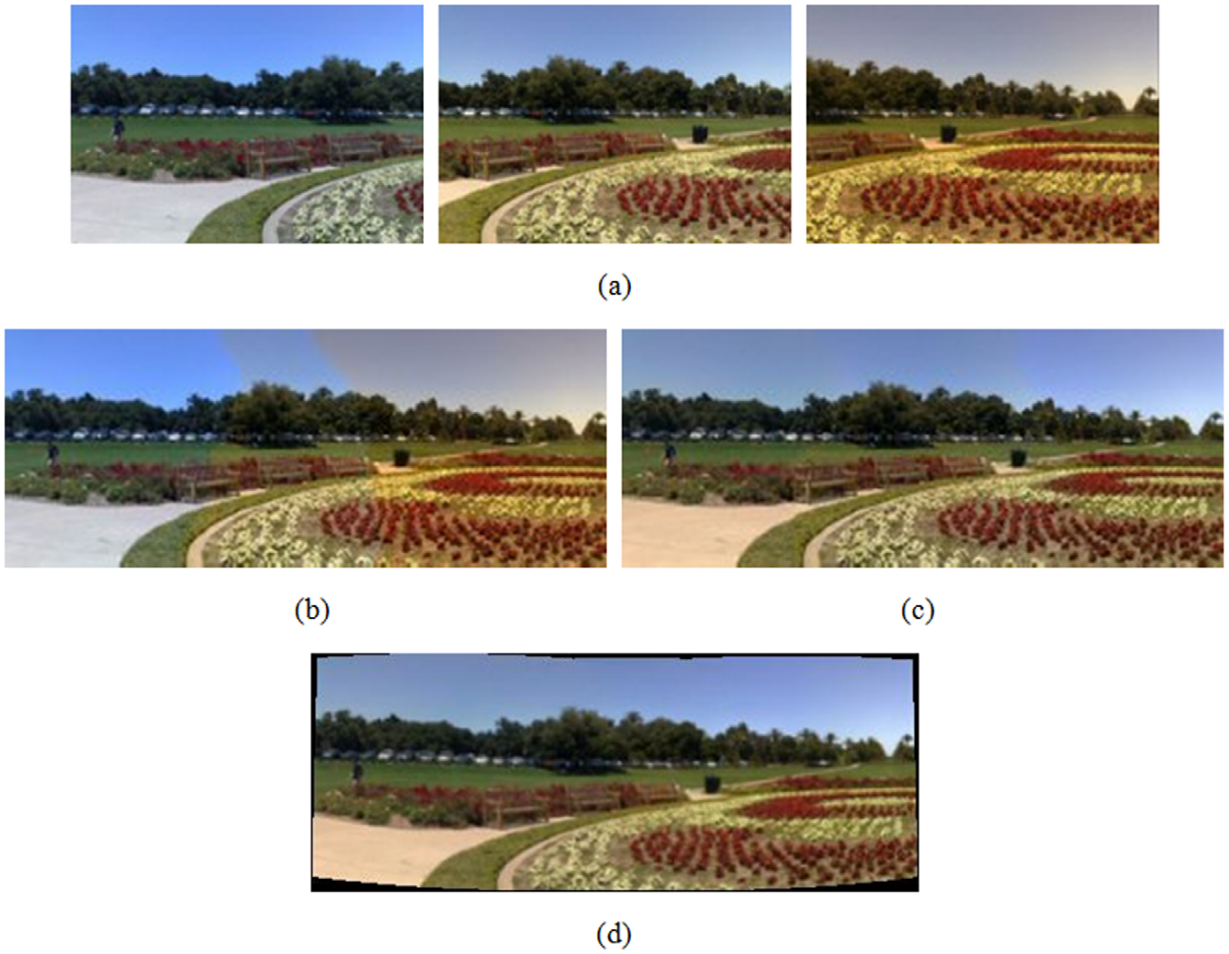
Image registration with vignette compensation and color difference adjustment, (a) three source images acquired from [**2**] with vignetting and color differences, (b), (c) stitched results without and with color correction by the fast stitching approach, respectively, (d) registered result with the proposed method.

**Figure 4:**
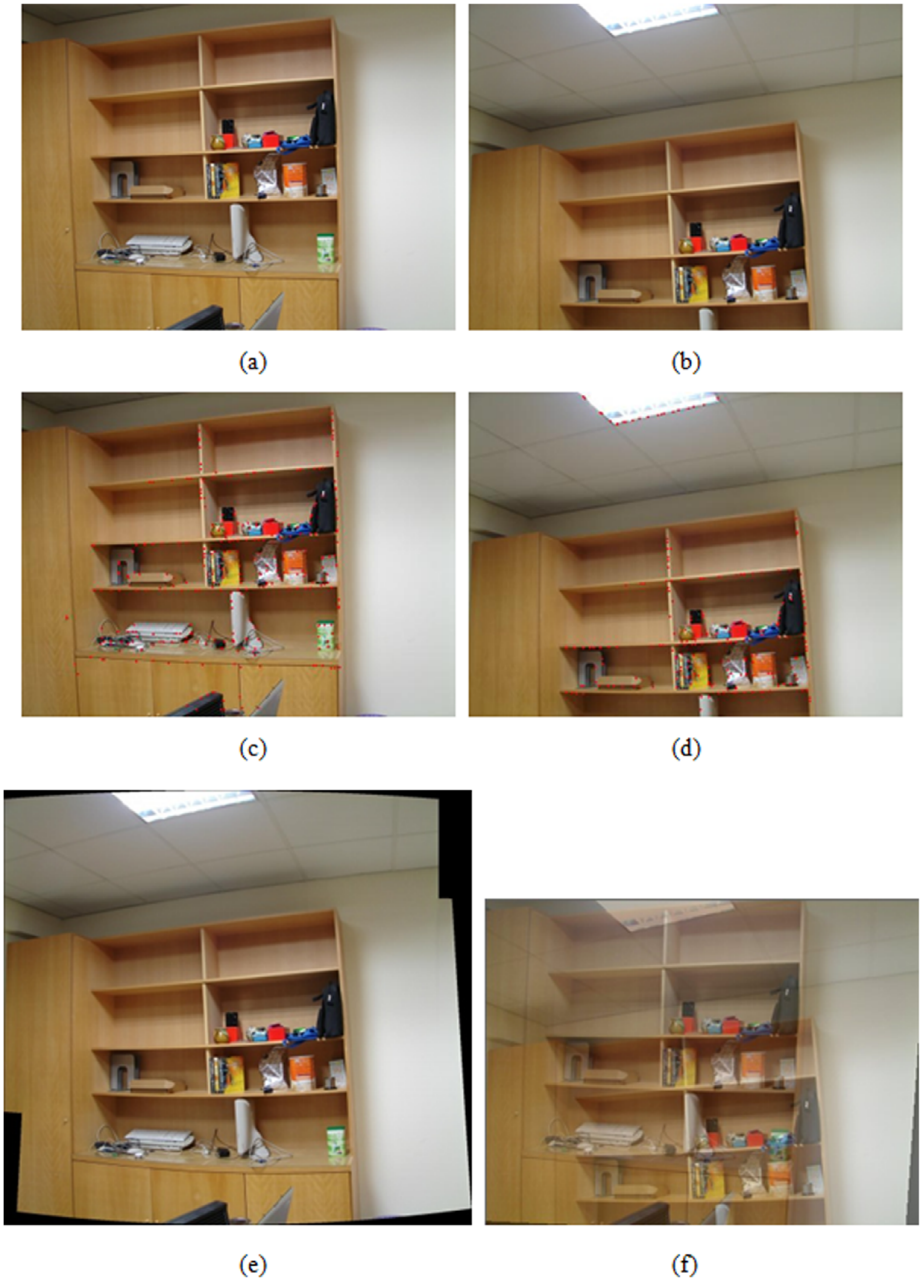
Registration result of indoor bookcases, (a), (b) an image pair used for registration, (c), (d) results of feature point extraction from (a) and (b), respectively, (e) registered result with the proposed algorithm, (f) registered result by optical flow-based approach.

**Figure 5:**
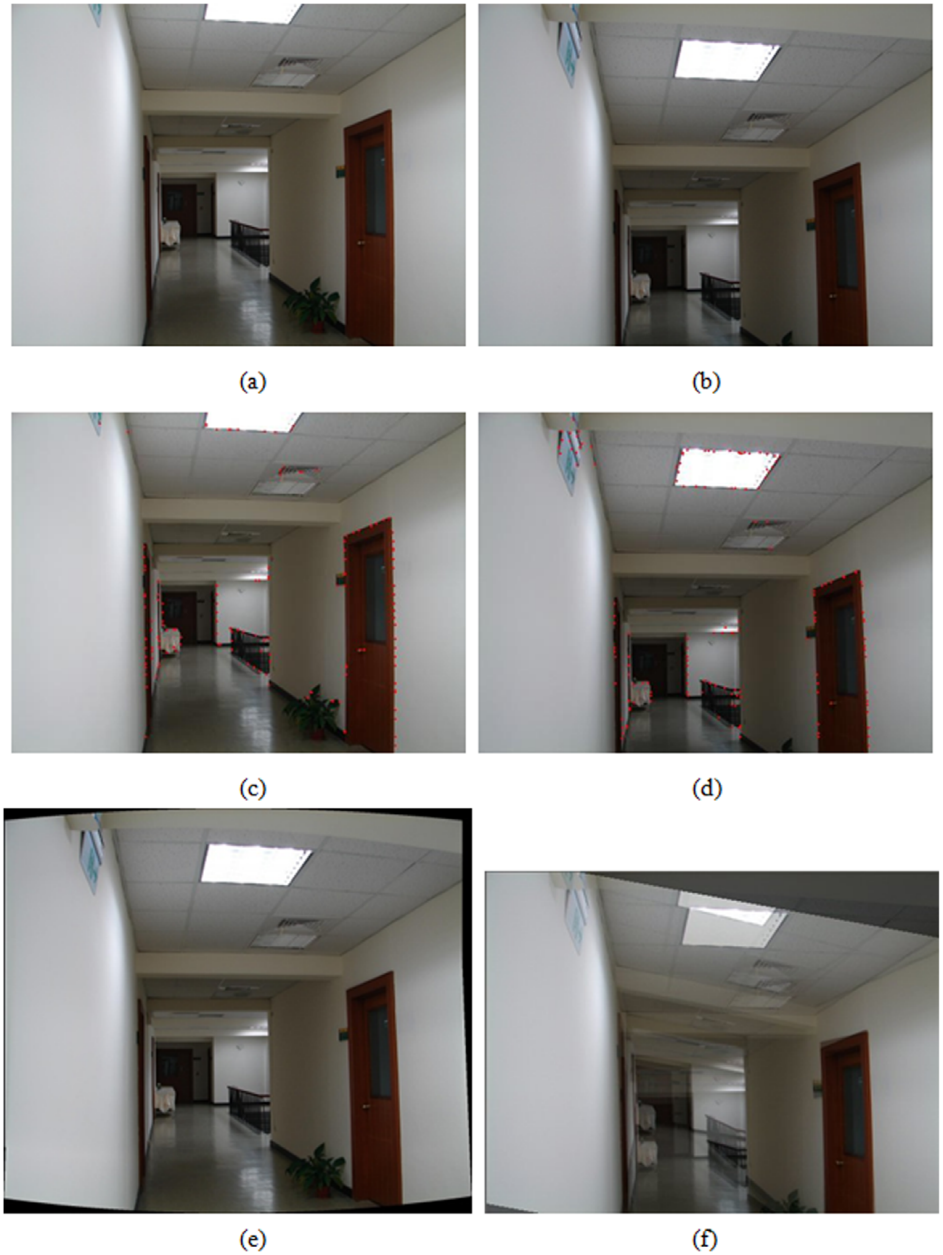
Registration result of office corridor, (a), (b) an image pair used for registration, (c), (d) results of feature point extraction from (a) and (b), respectively, (e) registered result with the proposed algorithm, (f) registered result by optical flow-based approach.

### 2.1. Feature Extraction

It is an important issue for feature-based image registration to extract significant features from acquired images, which will produce a great influence on the registration accuracy. More specifically, feature extraction is to extract representative features from the adjacent images, so as to effectively provide the geometrical and photometric information for image registration. Multi-resolution image decomposition is a useful technique for analyzing image information at various scales. Therefore, wavelet-based edge correlation, which had been verified the efficacy in feature extraction in our previous work [19], is used to extract the feature points with strong and consistent responses under different scales within a local area. In other words, feature points usually have larger values on the product of gradient moduli from multiscales, while the noise does not.

**Figure 6:**
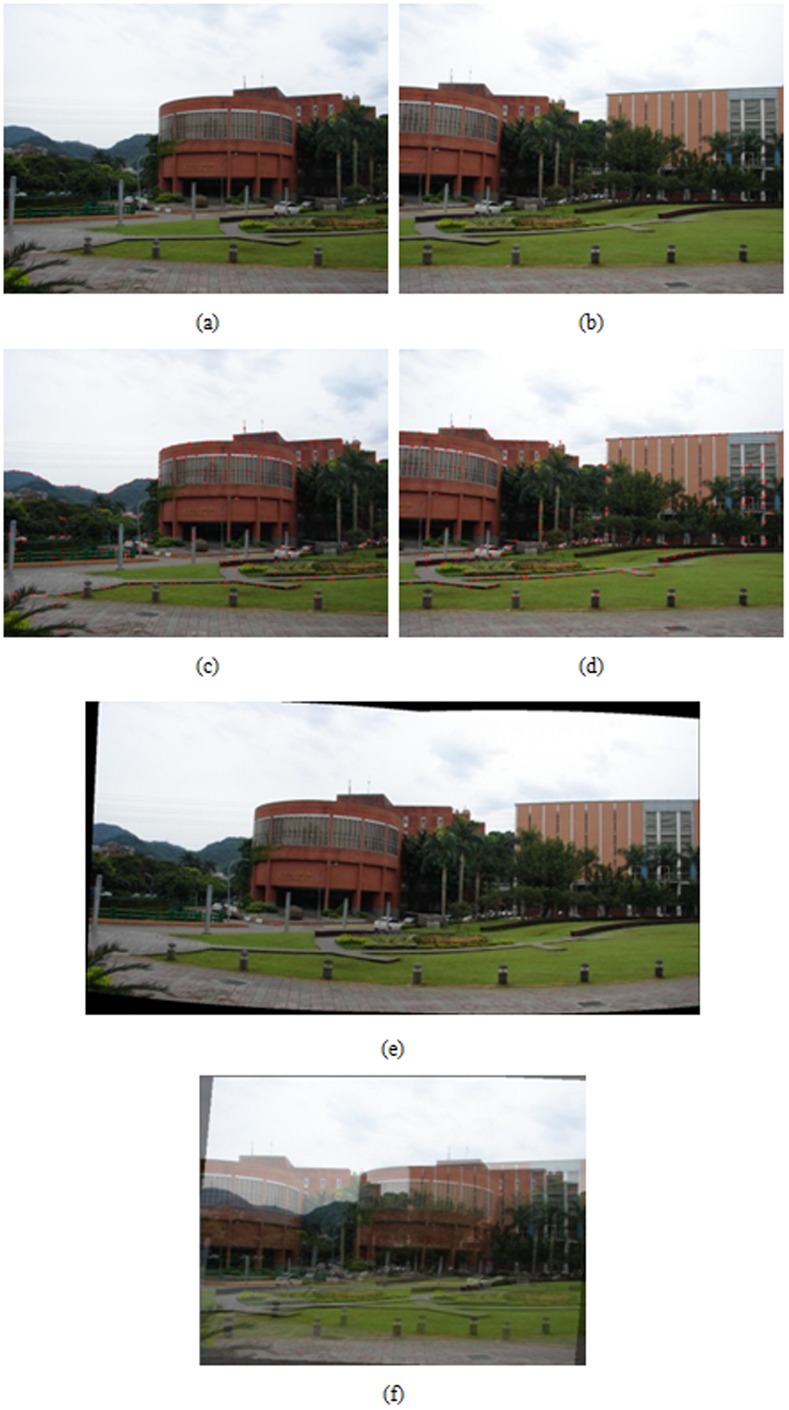
Registration result of building at a far distance, (a), (b) an image pair used for registration, (c), (d) results of feature point extraction from (a) and (b), respectively, (e) registered result with the proposed algorithm, (f) registered result by optical flow-based approach.

**Figure 7:**
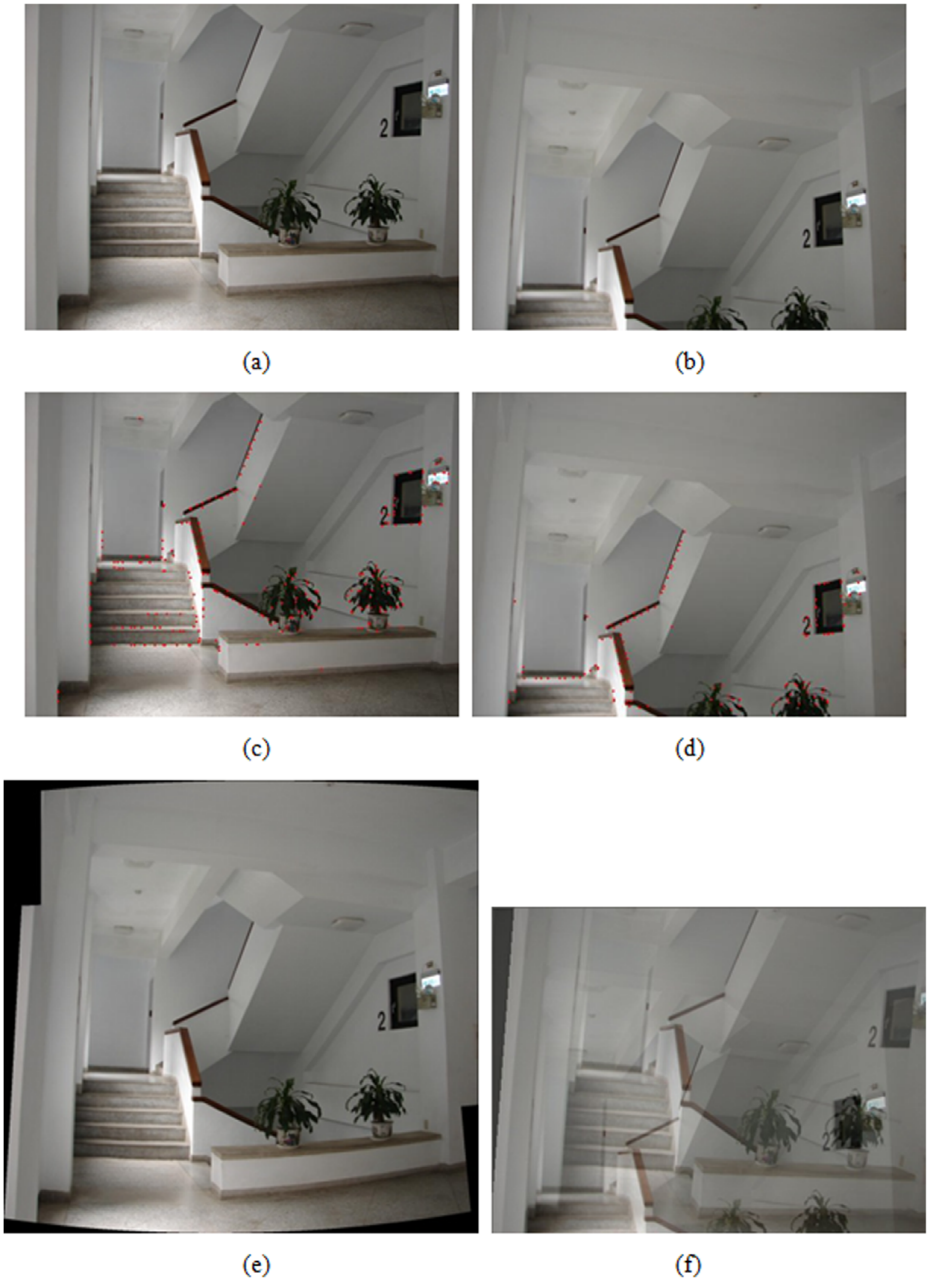
Registration result of indoor stairs, (a), (b) an image pair used for registration, (c), (d) results of feature point extraction from (a) and (b), respectively, (e) registered result with the proposed algorithm, (f) registered result by optical flow-based approach.

Due to the separable characteristic of wavelet transform, we represent 2D wavelet transform as two 1D ones in *x* and *y* directions, respectively,


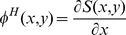
 and
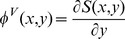
(1)where 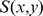
represents a 2D smoothing function. We denote 
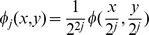
 as a dilation function of 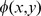 by a scaling factor *j*. The gradients 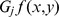 of an image 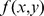 in the *x* and *y* directions and its modulus 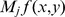 at level *j* are described as follows,
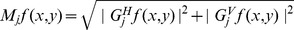
(2)where



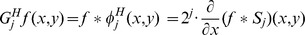


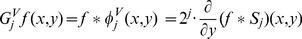
(3)All the edge points in image 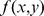 at level 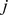 is located with local maxima of 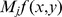. The edge correlation, which filters out the noise by a multiscale edge confirmation to detect reliable feature points, is represented as
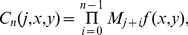
(4)where n is the level number, and j is the initial level. The true feature points can be obtained by means of edge correlation. Features and noise sometimes coexist in the wavelet domain, but features can usually exist in multiscales while noise can not [19]. In this study, the observed property is used to distinguish the true feature points from noise. The procedure of feature extraction is shown in Fig. 2. A test image is given in Fig. 2(a). Fig. 2(b) shows 2D two-level wavelet decomposition for the test image. The gradient modului of test image at level 1 and 2 are illustrated in Fig. 2(c) and 2(d), respectively. Finally, Fig. 2(e) shows the result of feature point extraction.

**Table 1:** Geometric transformation *T*s of four image pairs (IP) from Fig. 4, 5, 6, 7.

*T*	*T_11_*	*T_12_*	*T_13_*	*T_21_*	*T_22_*	*T_23_*	*T_31_*	*T_32_*	*T_33_*
IP 1 (Fig. 4)	0.982	0.003	−145.0	−0.010	0.999	−38.5	0.189	0.039	0.981
IP 2 (Fig. 5)	0.995	0.002	−76.8	−0.003	1.000	−6.5	0.100	0.007	0.995
IP 3 (Fig. 6)	0.999	−0.053	−5.9	0.052	0.925	385.0	−0.013	−0.376	0.927
IP 4 (Fig. 7)	0.982	0.023	−145.1	−0.015	0.999	43.2	0.190	−0.039	0.981

**Table 2:** Comparison of average registration quality from several (16) synthetic DSC images in terms of *SSD* and *CC* for three registration algorithms.

Registration Quality for Synthetic Images		*SSD* (mean±standard deviation)	*CC*
Result after	Optical Flow-based Motion Approach	78.19±9.78	0.541
	TPS-RPM	36.72±6.83	0.708
	Proposed Algorithm	12.10±3.13	0.896

**Table 3:** Comparison of average registration quality from all the pairs of DSC images in terms of *SSD* and *CC* for three registration algorithms.

Registration Quality for Real Images		*SSD* (mean±standard deviation)	*CC*
Results after	Optical Flow-based Motion Approach	127.63±19.47	0.291
	TPS-RPM	31.64±5.07	0.766
	Proposed Algorithm	10.43±2.24	0.923

### 2.2. Image Registration

The RPM algorithm was first proposed by Chui and Rangarajan [20]. It is a robust method for point-based registration, but it is somewhat slow in parameter convergence. In this study, we propose an analytic algorithm, namely analytic RPM (ARPM), to fast achieve image registration of perspective projection.

There are two point-sets 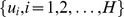 and 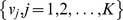 extracted from adjacent images, and the correspondence mapping is denoted by a matrix *M* consisting of *m_ij_*. The entire energy function minimized by the ARPM algorithm is as follows,
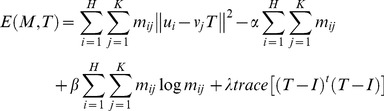
(5)where 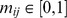 and it subjects to



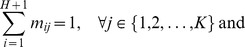


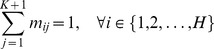
(6)The size of matrix *M* is 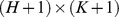 and its inner 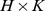 portion indicates the correspondence information for two point-sets. If a point *u_i_* corresponds to a point *v_j_*, then the entry *m_ij_* of the correspondence matrix *M* is equal to 1; otherwise, it is assigned to zero. In addition, in order to take the outliers into account so as to still hold the constraints of the row and column summation to one, an additional row and column is appended to the suffix of the correspondence matrix *M*.

All the components of the energy function are interpreted in turn in the following: The first term 
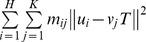
 is the error term that describes a corresponding problem by means of the 3-by-3 perspective projection *T*, which is the transform model of eight parameters, that is 
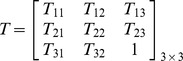
. It is a desirable transformation since the rotation, scaling, translation, and global shear are all taken into account. The second term 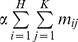 with the weighting 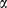 is used to avoid excessive null correspondence. If 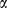 is large, fewer points are discarded as outliers. The third term 
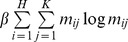
 with the temperature parameter 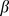 is an entropy function that guarantees the correspondence matrix *M* against negative numbers. The final term 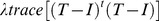 with the weighting 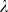 is a constraint on the geometric transformation *T* by means of the penalty on the remainder of subtracting the identity matrix *I* from the geometric transformation *T*.

As mentioned above, the minimization problem in equation (5) mainly consists of two related sub-problems: the point-sets correspondence and the geometric transformation between two adjacent slices. Given the point-sets correspondence, the geometric transformation can be evaluated by resolving the constrained least-squares problem. Given the geometric transformation, the point-sets correspondence is found and achieved by resolving the linear assignment problem. Inspired by the idea, the algorithm incorporates the update scheme by alternating the update of the correspondence and the transformation parameters while keeping the other fixed; it is expected to jointly improve the two solutions as well as finally converge to the optimal solution.

The registration algorithm mainly consists of two principal steps. It is accomplished by using an alternating update scheme. The first step is to update the point-sets correspondence matrix *M* as well as make sure that *M* corresponds to the row and column summation constraints all the time by keeping *T* fixed, with its currently evaluated transformation. Afterward, the solution for correspondence matrix *M* could be calculated by means of the differentiation of the energy function in equation (5) with respect to *m_ij_*,
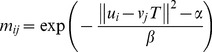
(7)The second step is to update the parameters of geometric transformation *T* with the correspondence matrix *M* held fixed. We propose an analytic ARPM approach to evaluate the parameters of *T* by means of the differentiation of the energy function in equation (5) with respect to *T_pq_*,
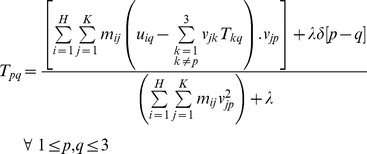
(8)where 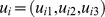, 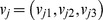, and 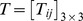. 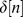, the unit sample sequence, is defined: 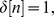 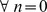; otherwise, 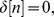 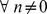. The two steps are iteratively performed while the temperature parameter 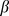 as well as the weighting 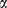 is gradually decreased. The decreasing process for the temperature parameter 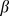 is similar to the deterministic annealing procedure [21]. The deterministic annealing with the temperature parameter 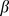 is a procedure to adjust the flexible degree of the correspondence matrix M. The correspondence matrix M eventually approaches a binary-values matrix as the temperature 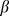 is gradually annealing. In addition, due to the fact that deterministic annealing can escape from the local minima, the approach is guaranteed to obtain the near-optimal solution.

### 2.3. Registration Refinement

After image registration, a FMLM method is proposed, which is extended from our previous work [19], to enhance registration results to further achieve subpixel precision. The modified algorithm is better than the conventional one in efficiency because the optimization problem is reformulated so that the Hessian matrix is no longer repeatedly calculated. In addition, the proposed feature-based method is efficient since the estimate of geometric transform is from only feature points, not the whole image. It greatly reduces the computation cost and enhances the robustness of registration as well. The FMLM method converges much faster than most other methods due to its nonlinear characteristic. The sums of square intensity errors 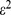 for feature-point blocks are minimized to measure the similarity between adjacent images. It is used as the measure of convergence and is defined,
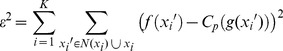
(9)

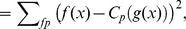
where *N*(*x_i_*) represents the neighborhood of the feature point *x_i_*, *K* stands for the number of feature points, *C_p_* is the geometric transform between *f*(*x*) and *g*(*x*) for mosaicing, and *fp* represents all the feature points and their neighboring points. For the adaptive geometric transform *C_p_*(.), we consider the general transform parameterized by the translation vector 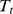, rotation angle 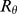, and scaling factor 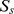. The geometric transform is represented by means of the operators,



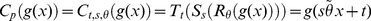
(10)The sum of square errors is then derived from the FMLM algorithm,
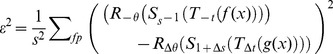
(11)

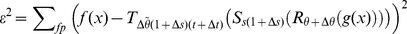
(12)The Hessian matrix *A* and gradient vector *b* are calculated,
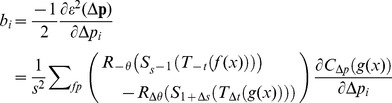
(13)and
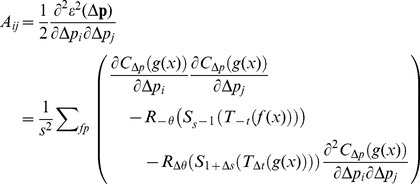
(14)where 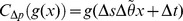. As the second term in 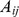 is small, it can be ignored.



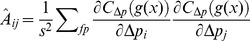
(15)Due to minimizing equation (11) with respect to 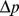 is the same as letting 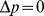 in equation (12) and then minimizing it with respect to 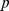. It is beneficial to calculate the Hessian matrix 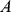 only once as the parameter 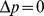. Hence, the gradient vector 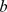 is rewritten as
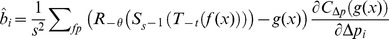
(16)


The parameter 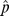 is then updated with estimated component 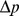 iteratively,
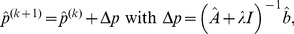
(17)where 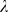 is a positive parameter. It is adjusted according to the convergent condition of errors. The FMLM method is updated iteratively until either the relative error is below a given threshold or the number of iteration reaches a predefined value.

### 2.4. *Vignette Compensation*

Vignette (lens vignetting or light fall-off) means a kind of light effect that light reaches the center of an image more than its edges. It makes the side areas of an image darker than its center area for a pure white image. Due to the optical nature of camera, images acquired from the camera may suffer from the vignette. Hence, the registered image shows vignetting artifacts, greatly reducing the quality of results. In order to compensate the vignetting phenomenon, a Gaussian-like model is constructed,
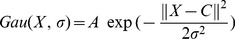
(18)where *X* represents a point in the overlapping zone, *A* is the maximum intensity, *C* is the coordinate of the image center point, and 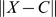 is the distance between *X* and *C*. For instance, let images 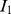 and 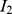 be adjacent and overlapping images. Points 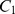 and 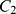 are the center point coordinates on images 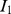 and 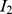, respectively. Point 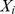 is a random point in the overlapping zone of images 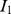 and 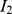, and its coordinates on images 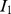 and 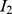 are 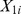 and 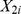, respectively. 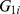 and 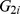 represent the intensity of point 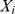 on image 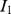 and 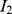, respectively. The relationship between these two images can be then written as:



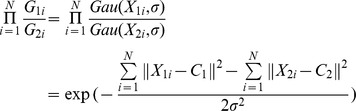



And,
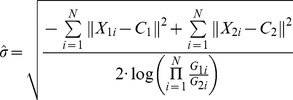
(19)where *N* is the number of overlapping points on image *I_1_* and *I_2_*, and 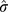 stands for the estimated standard deviation for vignette. Moreover, in order to suppress the influence of noise, 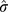 is calculated by using all the points in the overlapping zone instead of just a single point or a small image block. Finally, the intensities of all the points on image 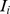 are recovered by removing the Gaussian-like degradation with 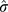:
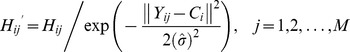
(20)where 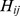 represents the original intensities with their corresponding coordinates 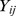 on image 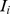, 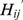 stands for the restored 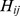, 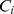 is the center point on image 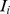, and M is the number of points on image Ii.

### 2.5. Adjustment of Color and Luminance Differences

The images are captured under automatic settings, including exposure, white balance, and focus. However, there are large color differences for exposure and white balance on adjacent images, when they are captured in the condition of illumination change. If the differences are not considered, it may make the registration unnatural. In order to adjust the color difference, the respective means of the RGB primitives for each pair of adjacent images in the overlapping region are calculated. The differences between the corresponding means are then accumulated from the left image pair to the right one sequentially. Finally, we adjust the color difference of all images according to the calculated accumulated difference. The equations with regard to the adjustment of color difference are described as follows,
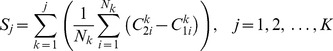
(21)

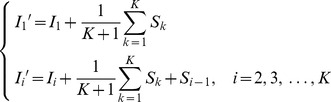
(22)where 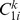 and 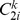 are the colors on adjacent images 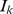 and 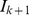, respectively, *N_k_* represents the number of overlapping points for image pair 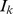 and 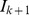, *K* is the number of image pairs, 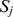
represents the color difference between images 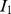 and 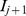, and 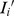 stands for the color-adjusted 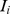. The results without and with vignette compensation and color difference adjustment are shown in Fig. 3. In addition, we also compare the results of the proposed method with that by other approach, which is referred to the fast stitching approach [2]. More specifically, Fig. 3(a) shows an example of three images acquired from [2] with vignetting and color differences. The stitched results, where the fast stitching approach is used, without and with color correction are shown in Fig. 3(b) and 3(c), respectively. Fig. 3(d) shows the registration result by the proposed method.

## Results and Discussion

### 3.1. Registration Examples

A hand-held digital still camera is used to capture images in the experiments. Each image is obtained with the resolution of 1024×768 pixels in 24 bit RGB format. A wide set of real image sequences are acquired and tested to evaluate the performance of the proposed algorithm by means of the visual quality assessment. The feature extraction and registration results for a variety of image pairs are shown in Fig. 4, 5, 6, 7. More specifically, subfigures (a) and (b) of each figure show the image pair that will be used for registration. The results of feature point extraction from subfigures (a) and (b) are shown in subfigures (c) and (d), respectively. Finally, the registration result of image pair with the proposed algorithm is shown in subfigure (e). The geometric transformation *T*s of these four image pairs from Fig. 4, 5, 6, 7 are listed in Table 1. An optical flow-based motion algorithm [22] is implemented for comparison. The algorithm is a registration technique that only takes affine transform into account. The registered results of the same image pairs for the optical flow-based motion algorithm are shown in Fig. 4(f), 5(f), 6(f), 7(f). The visual demonstrations indicate that the proposed method achieves better and finer results than the optical flow-based approach.

The setup of parameters in this experiment for the ARPM algorithm is described in detail as follows. The initial value for the temperature 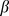 is assigned to slightly more than the longest distance of all point pairs, and it then gradually decreases with the annealing rate 0.93. The weighting 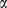 is assigned to 5. The point-sets correspondence *M* is initialized such that all the inner entries are 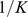 and the outlier ones are 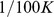. The geometric transformation *T* is initialized to a unit matrix. It is generally sufficient to achieve converged results by the alternating update on the correspondence *M* and geometric matrix *T* 20 runs.

Fig. 4 shows the image tilting problem for indoor bookcases with books and other stuffs inside. To show the importance of the proposed registration algorithm, the image pair is captured with a large vertical motion to make big difference. Fig. 4(e) is the result of the proposed registration algorithm, whereas Fig. 4(f) shows the results of optical flow-based approach. As seen in these two images, it can reveal that it is difficult for optical flow-based registration approach to handle large displacement problems. The results indicate that the proposed algorithm can resolve the panning and tilting problems to achieve accurate registration, while the optical flow-based approach still cannot. Fig. 5 shows the result of applying our registration method to the image pairs acquired from the office corridor with large overlapping and perspective distortion. Although the optical flow-based approach can track the matching points more accurate under the condition of small displacement, the result shown in Fig. 5(f) is still poor. The proposed algorithm can achieve accurate registration to overcome the distortion by considering perspective projection.

Fig. 6 show the registered results of image pairs acquired from the buildings with far distances. The displacements between the image pair are quite large in this case, so the optical flow-based approach cannot obtain precise registration. The results shown in Fig. 6(e) indicate that the proposed algorithm concerns large displacements of image pairs and distortions produced from the perspective projection of acquired images. Fig. 7 shows the results of registration for image pairs with large perspective distortion in tilting direction. The optical flow-based approach cannot register well since the perspective distortion is too large to accurately calculate the motion flow from matching points of image pairs. Fig. 7(e) reveals that the proposed algorithm can achieve satisfactory registration results even if the distortion in perspective projection is considerably large in titling direction.

### 3.2. Quality of the Registration

We have applied the proposed registration algorithm to all the image pairs. To assess the quality of the registration, we calculate the mean and standard deviation of the squared sum of intensity differences (*SSD*) as well as the correlation coefficient (*CC*)
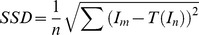


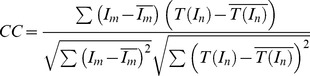
(23)where *I_m_* and *I_n_* represent a pair of images. *T*(.) is the geometric transform evaluated after each registration step. 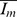 and 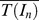 denote the average intensities. *n* is the pixel number within the overlapping zone. For each image sequence, the *SSD* and *CC* provide an indirect measure of registration quality.

We would expect in theory that the image difference only shows the underlying noise from image acquisition. However, the effects of the misregistration, geometric deformation are clearly visible in registered images. In the experiments, we compare the proposed registration method with the optical flow-based motion algorithm [22] and well-known thin-plate spline robust point matching (TPS-RPM) algorithm [20]. Table 2 and 3 summarize the average results of registration quality in terms of *SSD* and *CC* for these three registration algorithms for synthetic and real rat brain images, respectively. Table 2 lists the registration results for the synthetic images, which are selected from 16 DSC images with randomly selected parameters of geometric transform and Gaussian noise. The parameters for geometric transform *T* are selected in the ranges of 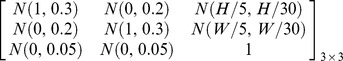, where *H* and *W* represent the height and width of images, respectively; the parameters for Gaussian noise are selected in the ranges of *N*(0, 2
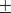
1). Table 3 lists the registration results evaluated from all the pairs of DSC images. The results indicate that the proposed algorithm can achieve satisfactory registration accuracy and quality, which is better than other two approaches.

### 3.3. Statistical Evaluation

To validate whether these three algorithms are significantly different or not, one-way analysis of variance (ANOVA) and multiple comparison tests are performed for the analysis of *SSD* and *CC* on both the synthetic and real data. The statistical analyses with one-way ANOVA are used to evaluate if the difference is significant for the factor *SSD* or *CC*. After analyzing with the one-way ANOVA, multiple comparison tests are used to estimate the *p*-values and significance of each pair of algorithms.

We obtain *p*-values less than 0.0001 and less than 0.0001 for *SSD* and *CC* respectively for synthetic data, while the *p*-values are less than 0.0001 and less than 0.0001 for *SSD* and *CC* respectively for real data. The results of test indicate that these three algorithms are significantly different among them. More detailed comparisons of *p*-values between each pair of approaches and multiple comparison tests of means are then performed. The results indicate that there are significant differences in the estimation of *SSD* and *CC* between the optical flow-based approach and proposed algorithm for synthetic data (*p*-values be <0.0001 and <0.0001 for *SSD* and *CC*, respectively). The results also denote that the proposed algorithm is significantly better than the TPS-RPM algorithm in both *SSD* and *CC* for synthetic data (*p*-values be <0.0001 and <0.0001 for *SSD* and *CC*, respectively). In addition to synthetic data, the results of tests for real data are also discussed. The results demonstrate that there are significant differences in *SSD* and *CC* estimation between the optical flow-based approach and TPS-RPM algorithm for real data, whereas they show that the proposed algorithm is significantly better than optical flow-based approach in *SSD* and *CC* estimation (*p*-values be <0.0001 and <0.0001 for *SSD* and *CC*, respectively). The results also indicate that the proposed algorithm is better than the TPS-RPM algorithm in both *SSD* and *CC* for real data (*p*-values be <0.0001 and <0.0001 for *SSD* and *CC*, respectively). Accordingly, the proposed algorithm obtains promising performance in the evaluation of registration quality for both the synthetic and real data.

### 3.4. Computation Time

The computation time is considered to evaluate the computational efficiency of the proposed algorithm in this study. We port the algorithm on mobile phones. More specifically, the registration results are obtained on a mobile phone with a 600 MHz processor and 384 MB RAM. The algorithm can also be performed on other mobile devices. The results indicate that the registration procedure takes 5.27±0.41 (mean ± standard deviation) seconds for image pairs acquired from Fig. 4, 5, 6, 7. It indicates that the proposed method is less time consuming in computation cost.

### Conclusion

In this study, we have presented an image registration algorithm for the application of consumer devices. A multiresolution wavelet-based method is exploited to retain significant features by discarding the noise. An analytic differential approach is then proposed to achieve fast registration of perspective projection. Finally, we refine registration accuracy to subpixel precision by the FMLM method. It reduces the computational cost quite significantly due to its feature-based and nonlinear characteristic. Moreover, we further improve image quality by performing vignette compensation and color/luminance difference adjustment. It shows that this study is fairly valuable for equipping the consumers with a powerful tool in life applications. In future work, the algorithm will be further improved to increase the robustness of registration.
